# The Frequency of Stoma-Related Readmissions After Emergency and Elective Ileostomy Formation: The Leicester Experience

**DOI:** 10.7759/cureus.73158

**Published:** 2024-11-06

**Authors:** Ting-Wei Wu, Wen Yuan Chung, Hui En Jewel Ng, Ashley Yap, Konstantinos Baronos, Deepak Paul, Christopher P Neal, David Bowrey

**Affiliations:** 1 General Surgery, University Hospitals of Leicester NHS Trust, Leicester, GBR

**Keywords:** colorectal malignancy, emergency colorectal surgery, ileostomy, ileostomy complications, open colorectal surgery, stoma related complications

## Abstract

Background

Approximately 9,000 patients undergo ileostomy formation each year in England. This includes those formed in both the elective and emergency settings. Recent studies have indicated a stoma-related complication rate of up to 83%.The aim of the current study was to ascertain the most common indications for ileostomy formation and to determine whether there were differences in the complication rates depending on whether the surgery occurred in the emergency or elective setting.

Methods

The study was a retrospective audit conducted at the University Hospitals of Leicester. Inclusion criteria were patients who underwent ileostomy formation between January 1 and December 31, 2023.

Results

The study cohort comprised 97 patients. Eleven of 44 (25.0%) patients in the emergency cohort had stoma-related complications, with their main reasons being high output stomas and bowel obstruction, which is higher compared to the elective patient cohort which had a complication rate of 5/53 (9.4%), with their main reasons being parastomal hernias and stoma prolapse. Ileostomies formed as a result of colorectal malignancy also led to a higher complication rate.

Conclusion

Our study suggests that ileostomies formed in the emergency setting as well as those formed due to colorectal malignancies had a higher rate of stoma-related complications. Stoma-related complications continue to be a common presentation to the emergency department.

## Introduction

Ileostomies are a potentially life-saving procedure, often formed in both the elective and emergency settings. Common indications include protecting a distal anastomosis after the defunctioning of the bowel [1], allowing diverted stool evacuation in cases where the rest of the colon has been removed (in conditions such as inflammatory bowel disease and colorectal cancer), or relieving bowel obstruction. Around 9,000 patients undergo an ileostomy formation each year in England [2]. Reported stoma-related complication rates in the literature have been variable, ranging from 10% up to 83% [3,4].

Early complications include skin irritation due to the high alkaline and active enzymatic content of the stoma output, necrosis of the stoma, or a high output (more than 1,500 ml per day [4]), which can go on to cause electrolyte imbalance and renal failure. Late complications on the other hand include the development of parastomal hernias, prolapse and stenosis.

Whilst some of the stoma-related complications such as skin irritation and a high output can be managed well with conservative measures [5], others may require surgical intervention which may go on to have long-lasting negative effects on a patient’s physical and mental wellbeing. Not only does this increase patient morbidity, it is also a major source of healthcare resource consumption. A retrospective study conducted by Brady et al. [6] revealed that patients with ostomy-related complications had a median of 13 inpatient admission days, 12 outpatient contacts within the first year and a mean cost of over £20,000 per patient. Over 90% of those costs were attributed to ostomy-related healthcare interactions, such as dealing with the most common surgical complications (high stoma output, peristomal skin complications and bleeding), and challenges with ostomy management such as leakage issues.

Most studies in current literature seem to suggest that stomas formed in the emergency setting are more likely to result in a higher complication rate [7] compared to those formed in elective settings. The long-term complication rate for ileostomies were also reported to be as high as 76% [8]. However, there is relatively little number of studies looking at emergency versus elective ileostomies specifically. As such, it was our aim to ascertain whether there were differences between these two cohorts, and what could be done to reduce these complication rates.

## Materials and methods

This study was a retrospective audit conducted at the University Hospitals of Leicester, United Kingdom. The Institutional Review Board of the University Hospitals of Leicester NHS Trust granted approval for this audit with the number #12921.

Primary objectives

To establish the most common indications for ileostomy formation in the emergency versus the elective setting and to assess the rate of readmissions due to stoma-related complications in the early (<30 days) and late (≥30 days) phase.

Secondary objectives

To compare whether there is a difference in the rate of stoma-related complications depending on the setting in which it was formed, as well as the indication. To determine the nature of the most common stoma-related complications and to grade them using the Clavien-Dindo Classification.

Inclusion and exclusion criteria

Our sample size was decided based on the periods of cohort. We aimed to establish the rates of ileostomy-related complications as it is a common presentation to the emergency department. As such, the study population comprised adult patients (aged 18 years or more) who underwent an ileostomy formation between January 1 and December 31, 2023 at the University Hospitals of Leicester NHS Trust.

Patients who had any other stoma formed, for example, colostomies or urostomies, were excluded, as were patients with missing data. Any patient who did not have their stoma formed in the year of 2023 or who had passed away during this time period for non-stoma-related reasons were also excluded. Patient follow-up was for a minimum of 12 months.

Data collection

After retrieving our population sample, all data was recorded onto an excel spreadsheet, including patient identification number, age, sex, BMI, admission and discharge date, comorbidities, primary surgical procedure and indication, type of ileostomy formed, whether it was formed in the elective or emergency setting, readmissions within or after 30 days, reason for readmission, type of stoma-related complication and date of readmission and discharge.

Statistical analysis

All data were entered into an Excel database (Microsoft, Redmond, Washington, United States), and analysis was performed using IBM SPSS Statistics 26. Descriptive statistics consisted of the mean and standard deviation for parametric distributions. Comparison amongst groups were performed with the T test for parametric variables. P values inferior to 0.05 were considered statistically significant. A Kaplan-Meier curve was used to estimate the stoma-related complication event rate over a 12-month period by plotting readmission rate against time.

## Results

Patient distribution

**Figure 1:**
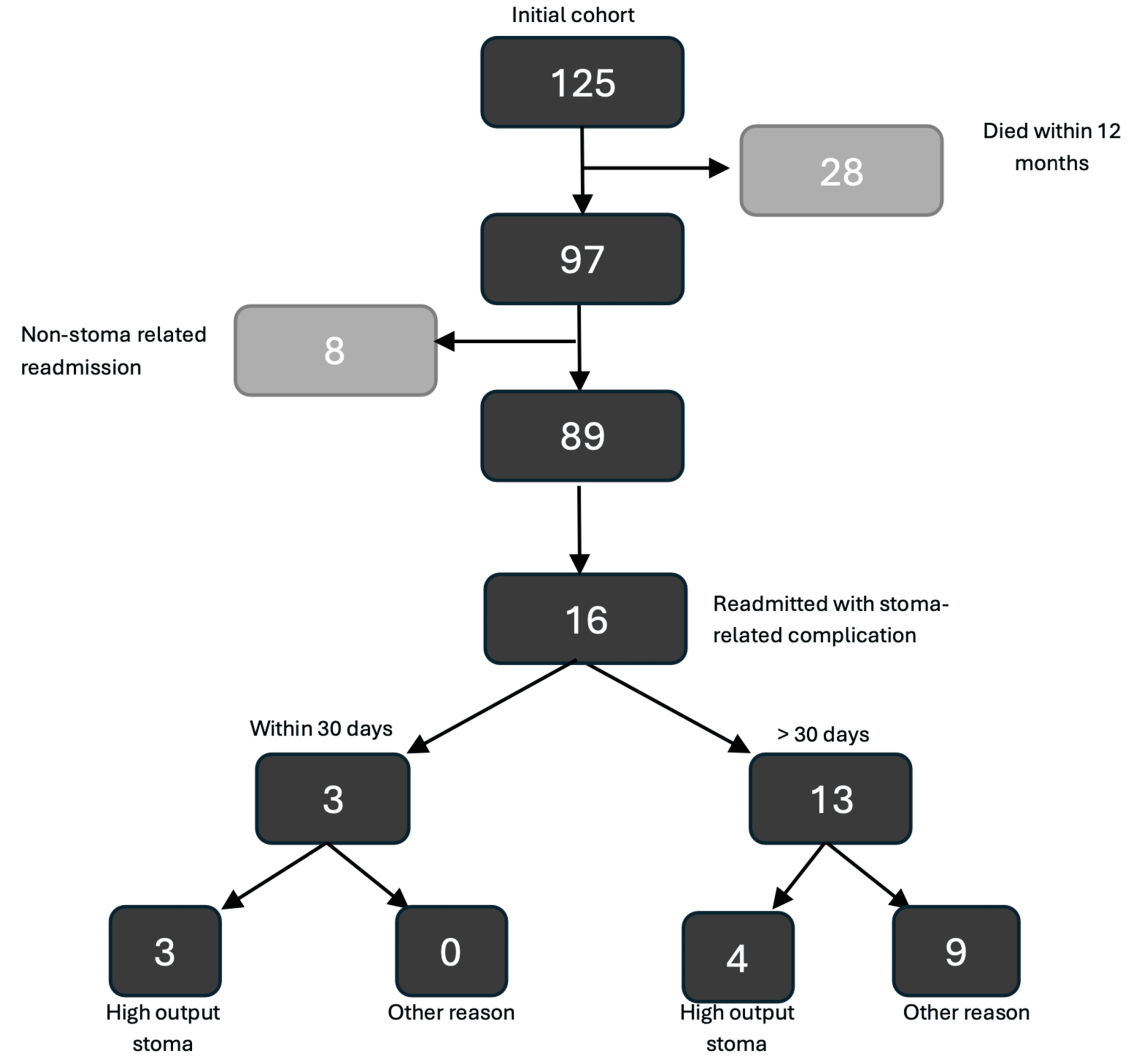
Flowchart demonstrating the readmission of patients with stoma-related complications

Figure 1 indicates patient disposition. Patients not surviving the first 12 months were excluded from the analysis (n=28). None of these patients experienced stoma-related readmissions or complications. Eleven patients died from advanced colorectal cancer, and 17 patients died from unrelated medical problems (urosepsis, aspiration pneumonia and pulmonary embolism) under the care of a non-general surgical specialty. There were 24 readmissions (16 stoma related, and eight non-stoma related).

Patient demographics and indications for ileostomy formation

Table 1 summarises the characteristics of those having an ileostomy on an elective versus emergency basis as well as the patient demographics. Within the emergency group of ileostomies formed due to colorectal malignancies, four were solely defunctioning loop ileostomies, five were resections with defunctioning ileostomies and four were resections and end ileostomies. For the elective group of the same indication, these numbers were two, 23 and 11, respectively.

**Table 1:** Patient demographics and Indications for ileostomy formation Values are presented as N(%). IBD: inflammatory bowel disease.

Patient Demographic	Emergency Cohort (n=44)	Elective Cohort (n=53)	P-Value*
Average age (mean years ± SD)	56 ± 17.3	56 ± 16.1	0.6
Average BMI (mean ± SD)	23.8 ± 4.03	27.7 ± 6.76	0.0035
Ratio of male:female	26:18 (59.1% vs 40.9)	33:20 (62.2% vs 37.8)	
*Student *t*-test was used for two-group comparison			
Indications			
Colorectal malignancy	13 (29.5%)	36 (67.9%)	
IBD	14 (31.8%)	9 (17.0%)	
Diverticular disease	4 (9.09%)	3 (5.66%)	
Small bowel obstruction (adhesional) causing gangrenous segment	4 (9.09%)	0	
Fistula	1 (2.27%)	0	
Stricture	1 (2.27%)	0	
Slow transit constipation	7 (15.9%)	5 (9.43%)	

Patient follow-up

**Figure 2:**
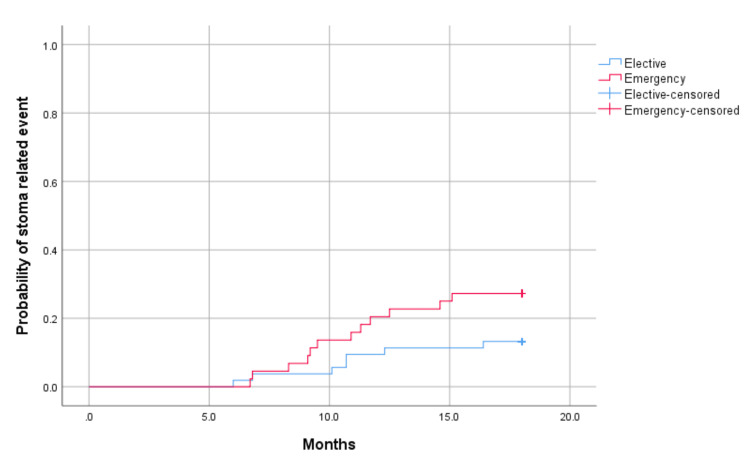
Kaplan-Meier graph showing stoma-related readmission rates: emergency versus elective

At a median follow-up interval of 10.7 months (range: 6 to 16.4), five of 53 (9.4%) of the elective and 11/44 (25%) of the emergency cohort had been readmitted, p=0.084 (Table 2, Figure 2).

**Table 2:** Stoma-related readmissions in the emergency versus elective cohort The values are presented as N(%).

Stoma-Related Readmission	Emergency Cohort (n=11)	Elective Cohort (n=5)
High output stoma	3 (27.3%)	0
Infection, abscess/parastomal cellulitis	1 (9.1%)	0
Obstruction at stoma site	4 (36.4%)	1 (20.0%)
Stoma prolapse	3 (27.3%)	2 (40.0%)
Parastomal hernia	0	2 (40.0%)

In both groups, a higher proportion of patients who were readmitted with stoma-related complications had their ileostomy originally formed due to reasons of a colorectal malignancy: six (54.5%) in the emergency cohort and three (60.0%) in the elective cohort. In both groups, all of these malignancy-related stomas were formed as either a purely defunctioning loop ileostomy or as a part of a resection and defunctioning ileostomy (emergency cohort (n=6), elective cohort (n=3)). None of the malignancy-related readmissions had their stomas formed originally as a resection and end ileostomy.

Patients with these malignancy-related ileostomies had readmissions due to bowel obstruction, prolapse, infection, parastomal hernias and high output. Using the Clavien-Dindo Classification for the management of surgical complications [9], the complications leading to stoma-related readmissions were graded. Most cases were either grade IIIb (n=5) or grade I (n=5). Four cases were of grade II, and two were of grade IIIa. A Kaplan-Meier curve was used to estimate the complication rates of the elective and emergency cohorts, by plotting readmission rate against time. There was a 16.5% (n=16) stoma-related complication readmission rate at a median follow-up time of 10.7 months.

## Discussion

According to our study, ileostomies formed in the emergency setting have a higher stoma-related complication rate compared to the elective setting. This may be because, in the emergency setting, the bowel is much more oedematous and inflamed, making the formation of a stoma much more technically difficult [10].

The most common reasons for stoma-related readmissions in the emergency cohort of our study were due to a high output stoma and bowel obstruction. Stomas formed from midline laparotomies in emergency colorectal surgeries are associated with a higher risk of high output stomas compared to elective surgeries performed laparoscopically [11]. This association may be explained by the fact that in the emergency setting, the bowel often would have been distended and or obstructed for some time, prior to the patient’s presentation. For patients who had their stomas formed due to inflammatory bowel disease, the high output may be explained by the association with an altered permeability of the bowel [12], due to reduced tight junction proteins. Damage to the tissue from trauma can also predispose to pro-inflammatory cytokine release, leading to intestinal permeability impairment.

It is possible that a higher BMI may predispose to complications of parastomal hernias and prolapse, as we found the incidence of this was higher in our elective cohort which had a higher average BMI of 27.7, compared to the emergency cohort whose average BMI was 23.8. This is in agreement with a study by Aida et al. [13] which found that high subcutaneous fat area was an independent risk factor for parastomal hernias. This may be explained by the fact that excessive adipose tissue may stimulate cytokines, leading to local inflammatory lesions which may lead to wound protraction during the healing process. Another study by Yamada et al. [14] suggested that fascial closure may be inhibited by excessive subcutaneous fat and a high BMI.

In terms of comparing our study with studies in the current literature, our stoma-related complication rate of 16.5% (n=16) over a median follow-up period of 10.7 months sits in the range of reported complication rates. A comparison table with similar studies is shown in Table 3.

**Table 3:** Our study results compared to similar studies in current literature

Study and year	Sample size	Type of stomas	Complication rate and most common reasons for readmission			Follow-up time period
			Emergency		Elective	
Our study, 2024	97	Ileostomies	25.0% obstruction, high output	9.4% hernia, prolapse		12 months
Ayik et al., 2024 [15]	872	Ileostomies and colostomies	63.6% mucocutaneous separation	69.6% dermatitis		30 days
Qureshi et al., 2018 [12]	195	Ileostomies and colostomies	48% ischaemia, retraction	25% ischaemia, retraction		12 months
Caricato et al., 2007 [16]	132	Ileostomies and colostomies	60% overall: dermatitis, hernia			4 months
Arumugam et al., 2003 [17]	97	Ileostomies and colostomies	50.5% overall: retraction, parastomal hernia			12 months

It can be seen in the current literature that the rate of stoma-related complications is widely variable, as are the types of complications seen. Current studies seem to suggest that stomas formed in the emergency setting have a higher complication rate compared to those formed in the elective setting, which is in agreement with our study.

If we compare complications rates in our study in terms of malignancy versus non-malignancy patients, those who had ileostomies formed due to colorectal malignancies had a higher complication rate in both cohorts, especially those who underwent cancer resection and loop ileostomy formation. Many of these patients who underwent resection also received adjuvant chemotherapy, and it is possible that this may have predisposed them to a higher risk of stoma-related complications. This finding is consistent with a study by Oliphant et al., [18] which found patients who underwent resection and loop ileostomy formation for colorectal cancer and had received adjuvant chemotherapy had a higher rate of stoma complications than those who did not receive chemotherapy.

Limitations

After excluding duplicate data entries, patients who passed away, and those who did not have their stoma formed in the year of 2023 or had any other stoma other than an ileostomy formed, our sample size was inevitably reduced. There is also a limited generalisability of our findings due to our study being conducted at a single centre.

## Conclusions

Ileostomies are commonly formed in both the emergency and elective settings. Patients often present with stoma-related complications after an ileostomy formation, and it appears that these complications are more common if the stoma was formed in an emergency setting compared to an elective setting. Ileostomies formed as a result of colorectal malignancy also appear to have a higher complication rate. Further research and a multi-centre study would be beneficial in increasing the generalisability of these findings, as well as reducing any potential bias. Stoma-related complications continue to be a common presentation to the emergency department. Therefore, it is important to identify the possible risk factors for the development of these complications and to emphasise the importance of stoma care so that we can help to better assess, manage and prevent these conditions.

## References

[1] Rajaretnam N, Lieske B (2024). Ileostomy. StatPearls [Internet].

[2] (2024). NHS Inform: Ileostomy. https://www.nhsinform.scot/tests-and-treatments/surgical-procedures/ileostomy.

[3] Mehboob A, Perveen S, Iqbal M, Moula Bux K, Waheed A (2020). Frequency and complications of ileostomy. Cureus.

[4] Babakhanlou R, Larkin K, Hita AG, Stroh J, Yeung SC (2022). Stoma-related complications and emergencies. Int J Emerg Med.

[5] Murken DR, Bleier JI (2019). Ostomy-related complications. Clin Colon Rectal Surg.

[6] Brady RR, Scott J, Grieveson S (2023). Complications and healthcare costs associated with the first year following colostomy and ileostomy formation: a retrospective study. J Wound Ostomy Continence Nurs.

[7] Dincer M, Çıtlak G (2019). Indications and complications of stoma formations in emergency surgery. Int J Inn Res Med Sci.

[8] Harris DA, Egbeare D, Jones S, Benjamin H, Woodward A, Foster ME (2005). Complications and mortality following stoma formation. Ann R Coll Surg Engl.

[9] (2024). BAUS: Grading of surgical complications. https://www.baus.org.uk/patients/surgical_outcomes/grading_of_surgical_complications.aspx.

[10] Miyo M, Takemasa I, Ikeda M (2017). The influence of specific technical maneuvers utilized in the creation of diverting loop-ileostomies on stoma-related morbidity. Surg Today.

[11] Assaf D, Hazzan D, Ben-Yaacov A, Laks S, Zippel D, Segev L (2023). Predisposing factors for high output stoma in patients with a diverting loop ileostomy after colorectal surgeries. Ann Coloproctol.

[12] Qureshi A, Cunningham J, Hemandas A (2018). Elective vs. emergency stoma surgery outcomes. World J Surg Surg Res.

[13] Aida T, Kamada T, Takahashi J (2024). High subcutaneous fat area is an independent risk factor for parastomal hernia after transperitoneal colostomy for colorectal cancer. J Anus Rectum Colon.

[14] Yamada T, Okabayashi K, Hasegawa H (2016). Age, preoperative subcutaneous fat area, and open laparotomy are risk factors for incisional hernia following colorectal cancer surgery. Ann Surg Oncol.

[15] Ayik C, Bişgin T, Cenan D, Manoğlu B, Özden D, Sökmen S (2024). Risk factors for early ostomy complications in emergency and elective colorectal surgery: a single-center retrospective cohort study. Scand J Surg.

[16] Caricato M, Ausania F, Ripetti V, Bartolozzi F, Campoli G, Coppola R (2007). Retrospective analysis of long-term defunctioning stoma complications after colorectal surgery. Colorectal Dis.

[17] Arumugam PJ, Bevan L, Macdonald L, Watkins AJ, Morgan AR, Beynon J, Carr ND (2003). A prospective audit of stomas: analysis of risk factors and complications and their management. Colorectal Dis.

[18] Oliphant R, Czerniewski A, Robertson I, McNulty C, Waterston A, Macdonald A (2015). The effect of adjuvant chemotherapy on stoma-related complications after surgery for colorectal cancer: a retrospective analysis. J Wound Ostomy Continence Nurs.

